# Population Structure, Historical Biogeography and Demographic History of the Alpine Toad *Scutiger ningshanensis* in the Tsinling Mountains of Central China

**DOI:** 10.1371/journal.pone.0100729

**Published:** 2014-06-23

**Authors:** Hongzhe Meng, Xiaochen Li, Penghai Qiao

**Affiliations:** Co-Innovation Center for Qinba Regions' Sustainable Development, College of Life Sciences, Shaanxi Normal University, Xi'an, China; University of Innsbruck, Austria

## Abstract

Population genetic structure, historical biogeography and historical demography of the alpine toad *Scutiger ningshanensis* were studied using the combined data mtDNA cytochrome b (cyt b) and the mtDNA cytochrome c oxidase subunit I (COI) as the molecular markers. This species has high genetic variation. There was a significant genetic differentiation among most populations. Three lineages were detected. The phylogenetic relationship analyses and the SAMOVA (spatial analysis of molecular variance) results showed significant phylogeographic structure. 82.15% genetic variation occurred among populations whereas differentiation within populations only contributed 17.85% to the total. Mantel test results showed a significant correlation between the pairwise calculated genetic distance and pairwise calculated geographical distance of the populations (regression coefficient  = 0.001286, correlation coefficient  = 0.77051, *p* (r_rand_≥r_obs_)  = 0.0185<0.05), indicating the existence of isolation-by-distance pattern of genetic divergence for cyt b + COI sequence, which suggests that the distribution of genetic variation is due to geographical separation rather than natural selection. The population expansion or contraction and genetic differentiation between populations or lineages could be explained by topography and the repetitive uplifts of the Tsinling Mountains and the climatic cycles during the late Pliocene and Pleistocene. *S. ningshanensis* experienced a rapid population expansion about 40,000 years before present. The current decline in population size was probably caused by anthropogenic disturbance. Current populations of *S. ningshanensis* are from different refugia though the location of these refugia could not be determined in our study. Topography, climatic changes and repetitive population expansion/contraction together led to the high level of genetic variation in *S. ningshanensis*. A total of three management units (MUs) was determined, which must be considered when conservation policy is made in the future.

Population genetic structure, historical biogeography and historical demography of the alpine toad *Scutiger ningshanensis* were studied using the combined data mtDNA cytochrome b (cyt b) and the mtDNA cytochrome c oxidase subunit I (COI) as the molecular markers. This species has high genetic variation. There was a significant genetic differentiation among most populations. Three lineages were detected. The phylogenetic relationship analyses and the SAMOVA (spatial analysis of molecular variance) results showed significant phylogeographic structure. 82.15% genetic variation occurred among populations whereas differentiation within populations only contributed 17.85% to the total. Mantel test results showed a significant correlation between the pairwise calculated genetic distance and pairwise calculated geographical distance of the populations (regression coefficient  = 0.001286, correlation coefficient  = 0.77051, *p* (r_rand_≥r_obs_)  = 0.0185<0.05), indicating the existence of isolation-by-distance pattern of genetic divergence for cyt b + COI sequence, which suggests that the distribution of genetic variation is due to geographical separation rather than natural selection. The population expansion or contraction and genetic differentiation between populations or lineages could be explained by topography and the repetitive uplifts of the Tsinling Mountains and the climatic cycles during the late Pliocene and Pleistocene. *S. ningshanensis* experienced a rapid population expansion about 40,000 years before present. The current decline in population size was probably caused by anthropogenic disturbance. Current populations of *S. ningshanensis* are from different refugia though the location of these refugia could not be determined in our study. Topography, climatic changes and repetitive population expansion/contraction together led to the high level of genetic variation in *S. ningshanensis*. A total of three management units (MUs) was determined, which must be considered when conservation policy is made in the future.

## Introduction

Population genetic structure refers to the geographical pattern of genetic diversity within or among populations. It could be influenced by gene flow, genetic drift, selection, mutation and recombination. Gene flow is caused by the movement of individuals from one population to another [1]. Estimation of the gene flow level allows conservation biologists to understand the relationships between populations and assess levels of genetic variation in order to evaluate the relative levels of conservation concern hierarchically across populations in a species. Genetic drift is the change in the frequency of a gene variant in a population due to random sampling [2]. http://en.wikipedia.org/wiki/Genetic_drift - cite_note-Masel_2011-1#cite_note-Masel_2011-1Genetic drift may lead to disappearance of gene variants and thereby reduce genetic diversity.

Phylogeography connects historical processes in evolution with spatial distributions [4]. Analysis of mitochondrial data promoted the empirical development of phylogeography [3]. The statistical phylogeography is one of the widely used approaches in phylogeography, which takes into account the stochasticity of genetic processes into demographic inference based on coalescent models for parameter estimation [4], [5].

**Figure 1:**
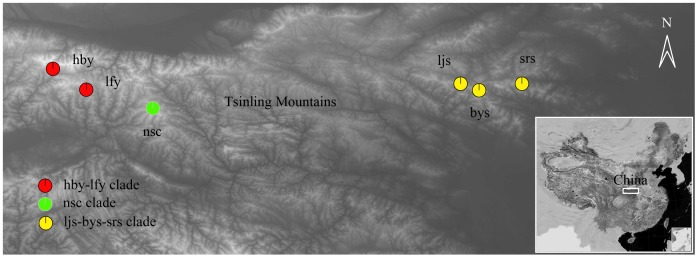
Locations of sampled populations and geographical distribution of *S*. *ningshanensis* clades on the Tsinling Mountains. Nsc is also the type locality of *S. ningshanensis*.

The Tsinling Mountains are located in the central part of China, stretching from west to east (Fig. 1). These Mountains are boundary between Oriental realm and Palaearctic realm according to the zoogeographical regions of China [6], and also the watershed for Yangtze River and Yellow River catchment areas, for climate, flora and fauna in China [7]–[9]. The Oriental and Palaearctic species congregate here forming a specific biotic province and containing rich animal and plant resources [10], [11]. Like other regions in the northern hemisphere, the Tsinling Mountains experienced several glacial-interglacial cycles during Pleistocene [12]–[18]. The climate associated with Pleistocene glacial cycles in East Asia was likely mild and characterized by a mosaic of mountains [19], [20]. The past climatic events, such as the Quaternary glaciation, are believed to have played an important role in forming the geographical pattern of the montane species and could leave the vestiges in geographical distribution of genetic diversity of population [21]–[26]. The founder effect during the postglacial population recovery causes a reduction in population genetic diversity [27], [28], and the subsequent rapid population expansion [29] may erase the previous geographical differences of the genetic diversity.

The alpine toad *Scutiger ningshanensis* was described from the western part of the Tsinling Mountains [30] (Fig. 1). Four years later, the second specimen of this species was collected from the same locality [31]. Since then, other specimen of this species was not collected until 2009 when some specimens were collected from several localities in the eastern part of the Tsinling Mountains [32]. Other than the reports on collection of additional specimens, only the biological characteristics of tadpoles of this species was studied [33]. The habitat of this species was roughly divided into two parts: the western part and the eastern part. Is this geographical pattern caused by habitat fragmentation or by populations from different glacial refugia? Does the isolation by distance between the local populations result in occurrence of any speciation events? The aims of the present study were to explore the population genetic structure, historical biogeography and the historical demography of *S. ningshanensis*.

## Materials and Methods

### Ethics statement

This study was approved by the Institutional Animal Care and Use Committee (IACUC) of Shaanxi Normal University. Only the clipped toes or tail tips of tadpoles were used for extraction of total DNA. No specific permissions were required for these locations/activities. This species was ranked “Endangered B2ab(iii)” in “The IUCN Red List of Threatened Species™ 2013” based on an outdated information that this species was only found at the type locality (nsc) [31] (http://www.iucnredlist.org). Actually, in addition to the type locality, this species was reported later in 2009 from a variety of localities including the Baiyunshan Mountains (bys), Shirenshan Mountains (srs) and Laojunshan Mountains (ljs) [32] (Fig. 1), which indicated that this species doesn't meet the criteria of critically endangered or endangered defined in The IUCN Red List of Threatened Species™ 2013, therefore this species should be removed from the red list. However, the new data was not taken into account when this species was ranked “Endangered B2ab(iii)” in “The IUCN Red List of Threatened Species™ 2013”. The specific location (GPS coordinates) of our study was given in Table 1.

**Table 1:** Sampling information and haplotypes based on cyt b and COI for 6 sampled populations of *Scutiger ningshanensis*.

Population	Location	n	GPS coordinates	Elevation (m)	Haplotypes
hby	Huangbaiyuan, Taibai Co., Shaanxi Prov.	18	33.8749N 107.5168E	1652	hby1 (1), hby10 (1), hby12 (4), hby13 (1), hby14 (1), hby15 (1), hby16 (1),hby19 (1), hby2 (1), hby20 (1), hby3 (1), hby4 (1), hby7 (1), hby8 (1), hby9 (1)
lfy	Liangfengya, Foping Co., Shaanxi Prov.	17	33.6668N 107.8529E	2047	hby13 (1), lfy1(1), lfy10 (1), lfy12(2), lfy13 (1), lfy14 (1), lfy15 (1), lfy16 (1), lfy17 (1), lfy2(1), lfy3 (1), lfy4 (1), lfy5 (1), lfy6(1), lfy7 (1), lfy8(1)
nsc	Pingheliang, Ningshan Co., Shaanxi Prov.	15	33.4744N 108.5253E	2000	nsc1 (1), nsc10 (1), nsc11 (1), nsc12 (1), nsc13 (1), nsc14 (1), nsc15(1), nsc2 (1), nsc3 (1), nsc4(1), nsc5 (1), nsc6 (1), nsc7(1), nsc8 (1), nsc9 (1)
ljs	Laojunshan, Luanchuan Co., Henan Prov.	18	33.7272N 111.6309	1590	bys4 (6), ljs1 (4), ljs10 (2), ljs11 (1), ljs18 (1), ljs4 (1), ljs5 (2), ljs6 (1)
bys	Baiyunshan, Songxian Co., Henan Prov.	16	33.6535N 111.8283E	1675	bys1(8), bys10 (1), bys11 (2), bys14 (1), bys15 (1), bys4 (1), bys7 (2),
srs	Shirenshan, Lushan Co., Henan Prov.	15	33.7286N 112.2542E	1642	ljs11 (5), srs1 (3), srs15 (1), srs16 (1), srs17 (1), srs18 (1), srs5 (1), srs6 (1), srs9 (1)

n, sample size.

### Sampling and laboratory protocols

Our sampling covers the entire known distribution of this species. Furthermore, to make an extensive sampling, we explored the whole Tsinling Mountains, and fortunately collected this species at two locations where the distribution of this species has not been recorded. A total of 99 samples were collected from 6 localities during 2011 and 2013 (Table 1, Fig. 1). Eight samples of the alpine toad *Scutiger boulengeri* were collected from Jone County (34.539922N 103.491647E), southern Gansu Province, China. *S. boulengeri* will be used as outgroup in phylogenetic relationship analysis.

The clipped toes or tail tips of tadpoles were preserved in 100% ethanol and stored at −20°C. A continuous fragment (1009 bp) of the mitochondrial cytochrome b (cyt b) was amplified using PCR (MyCycler Thermal Cycler), with primers FrogGlu-f 5′-TGATCTGAAAAACCACCGTTG-3′ and FrogThr-r 5′- CTCCATTCTTCGRCTTACAAG-3′ [34]. A continuous fragment (631 bp) of the mitochondrial cytochrome c oxidase subunit I (COI) was amplified using PCR (MyCycler Thermal Cycler), with primers forward LepF5′-ATT CAA CCA ATC ATA AAG ATA TTG G-3′ and reverse LepR5′-TAA ACT TCT GGA TGT CCA AAA AAT CA-3′ [35]. The PCR products were purified using a purification kit (DC3511-02/3514-02 250 Preps, Biomiga, USA). Sequencing reactions were carried out with the PCR primers using ABI Prism BigdyeTM Terminator Cycle Sequencing Ready Reaction Kit on ABI 3730XL sequencer. All sequences have been deposited in the GenBank databases under accession numbers KF757340–KF757391 (*S. ningshanensis* cyt b), KF757392–KF757439 (*S*. *ningshanensis* COI); KJ082065– KJ082072 (*S. boulengeri* cyt b), KJ082073–KJ082080 (*S. boulengeri* COI). Indicators of nuclear mitochondrial pseudogenes (numts), such as indels, stop codons and double peaks in sequence chromatograms [36] were not found.

### Determination of generation time

A skeletochronological study of longevity of *S. ningshanensis* was conducted. The clipped phalanges were stored in 95% ethanol solution. The phalanges were washed in running tap water, then decalcified in 3% nitric acid for 12 to 24 hours. The mid-diaphyseal region of the phalanges was cross-sectioned at 12–20 µm using a microtome and stained with hematoxylin for two min. Sections were examined under a light microscope and the number of lines of arrested growth (LAGs) was counted [37], [38]. The number of LAGs represents the age of the toad.

### Nucleotide polymorphism

The sequences were aligned with Clustal X1.83 [39]. The aligned sequences were edited using the program BioEdit 7.0.9.0 [40]. All analyses were performed based on the combined mitochondrial DNA data. Haplotype inference was conducted through Collapse 1.2 (http://darwin.uvigo.es). The number of variable and parsimony informative sites was determined using the program DnaSP 5.10.01 [41], and haplotype diversity (*H*d) and nucleotide diversity (*P*i) were determined through Arlequin 3.5.1.2 [42].

### Phylogenetic analyses

The substitution model selection was implemented in jModelTest 2.1.4 [43], the TIM2+I+G model was selected for all datasets by likelihood ratio tests either under the Akaike Information Criterion (AIC) or under the Bayesian Information Criterion (BIC). Bayesian inference (BI) was used to generate a phylogenetic hypothesis of the DNA haplotypes. BI was performed in MrBayes 3. 2 [44] with 1,200,000 generations, sampling trees every 100 generations. Two independent runs each with four simultaneous Monte Carlo Markov chains (MCMC) were conducted. The first 25% of generations were discarded as ‘burn-in’. The convergence of chains was confirmed until average standard deviation of split frequency is below 0.01 (0.009889) and the potential scale reduction factor (PSRF) is close to 1.0 for all parameters. In phylogenetic analysis *S. boulengeri* was used as outgroup.

### Population structure analyses

The population comparisons using pairwise difference as distance method, and the partition of genetic diversity within and among populations were analyzed by analysis of molecular variance (AMOVA) [45] using Arlequin3.5.1.2 [42] with 10,000 permutations. Mantel tests [46] were also conducted in Arlequin3.5.1.2 to assess the significance of isolation by distance (IBD) between populations with 10,000 random permutations on matrices of pairwise population *F*_ST_ values and the geographical distances. Pair-wise *F*_ST_ values between populations were estimated using Arlequin3.5.1.2, while geographical distances between populations were calculated online at http://www.gpsvisualizer. com/calculators#distance.

The spatial genetic structure of haplotypes was analyzed using the program SAMOVA1.0 [47] (http://web.unife.it/progetti/genetica/Isabelle/samova.html) with 1,000 permutations. The number of initial conditions was set to 100 as recommended by Dupanloup et al. [48]. The number *K* of groups of populations ranged from 2 to 4. The *K* with the highest *F*ct represents the best number of groups and the best population configuration.

### Historical biogeography

**Figure 2:**
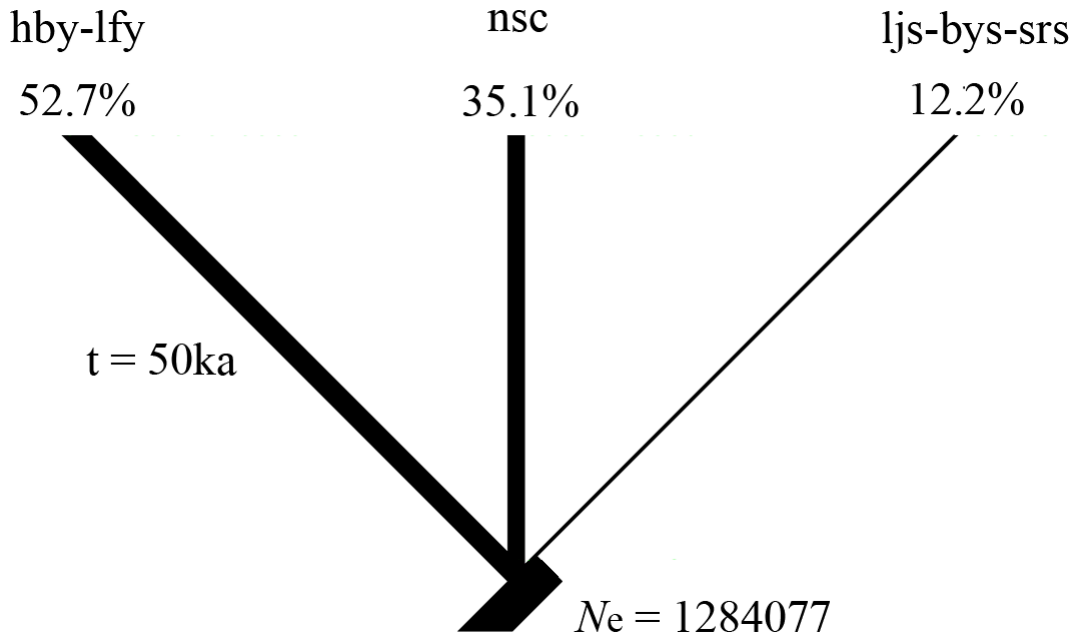
The model used to test the refugial hypotheses for *S. ningshanensis* using coalescent simulations. A single-refugium hypothesis concerning the refugia during the Dali glaciation (the last maximum glaciation in China which occurred about 50 ka before present) was tested. The detail interpretation for this model is given in the text. Branch lengths are time in generations based on a 6-year generation time in *S*. *ningshanensis*. Branch widths (effective population size, *N*_e_) are scaled for each group based on the proportion of the total *N*_e_ that each group comprised.

The effective population size (*N*_e_) of each clade (geographical group) for coalescent simulations was converted from Theta using the equation *θ* = *N*_e_µ with µ = 0.65×10^−8^ (per lineage per million years) ×6 (generation time of *S. ningshanensis*). The *θ*-values were estimated using maximum likelihood method in the program Lamarc2.7.5 [48]. Total *N*_e_ was the sum of the *N*_e_ for all groups and the proportion of total *N*_e_ that each group comprised were used to scale the branch width of hypothesized trees (models of population divergence) (Fig. 2) [49]–[51]. Branch widths can be controlled by the Adjust Lineage Widths tool (the horizontal ruler) in the Tree Window in the program Mesquite2.75 [52].

A single-refugium hypothesis of population divergence during the Dali glaciation (the last maximum glaciation in China which occurred about 50 ka before present) was tested using the maximum likelihood estimation implemented in Mesquite2.75 [52] to infer the distributional area of the most recent common ancestor (MRCA) of clades. The single-refugium model hypothesizes that all current geographical populations derived from a single refugium at the end of the Dali glaciation and that all population divergences were concurrent and resulted from the fragmentation of a widely distributed common ancestor's range (Fig. 2). Branch lengths are time in generations based on a six-year generation time in *S. ningshanensis*. The clades were treated as taxon states and each haplotype as character states. The haplotype trees by coalescence within a diverging population tree (model of population divergence) were simulated, and fit of a haplotype tree to a population tree was calculated to search for population trees that optimize fit of gene trees.

### Historical demography

Neutrality tests were calculated in Arlequin3.5.1.2 [41], Fu's *F*_S_ test [53] and Tajima's *D* [54] were used to detect evidence of recent demographic expansion within each inferred clade, under which negative value are expected [55]. Inference of population expansion events was performed using a mismatch analysis [56], [57] using Arlequin3.5.1.2 with the number of bootstrap replicates set to 10,000 to explore the demographic history of the studied populations. A recent growth is expected to generate a unimodal distribution of pairwise differences between sequences [56]. The validity of the expansion model was tested using the sum of squared deviations (*SSD*) and Harpending's raggedness index (*R*) between observed and expected mismatches.

The site-frequency spectrum (for segregating sites) was calculated in DnaSP5.10.01 [41] to detect the excess of singleton mutations. The null hypothesis of the neutral model (constant population size) was rejected when the allelic frequency spectrum for the entire population revealed an excess of singleton mutations. The excess of singleton mutations could be caused by the expansion [58].

Furthermore, the Bayesian Skyline Plot (BSP) approach was used to estimate the demographic history in BEAST v1.8.0 [59]. The log file was analyzed in Tracer. Strict molecular clock model was selected based on the results from Tracer. A mean mutation rate of 0.65% change per lineage per million years was used [60]–[67]. Two independent BEAST runs from the same XML file were carried out and then the log output files were combined using LogCombiner. The combined log file was analyzed in Tracer to see if the Trace for each parameter has converged well on a stationary distribution.

### Detection of cryptic species

To detect the existence of potential cryptic species, uncorrected p-distances [68], [69] for all lineage pairs were calculated in PAUP4.0 [70] from all sequences. The average p-distances of all possible pairs of sequences (every sequence pair contains sequences from different lineages) were calculated.

## Results

### Generation time

The average longevity of tadpoles was estimated by Lu et al. [33]. The whole longevity equals the longevity of adult plus the longevity of tadpole. The average duration of the tadpole stage of *S. ningshanensis* is three years [33] and the average duration of the adult stage after metamorphosis is six years. The average life span of *S. ningshanensis* is nine years. The average generation time (GT) of *S. ningshanensis* is six years.

### Genetic variation

A total of 107 samples were sequenced, including 99 samples of *S.ningshanensis* and eight of *S. boulengeri*. A total of 1009 base pairs of cyt b gene and 631 base pairs of COI gene was obtained, 67 haplotypes were identified for the combined gene sequences (cyt b+COI) of *S. ningshanensis*. Of the combined 1640 nucleotide sites, 181 were variable (polymorphic sites or segregating sites), 121 were parsimony informative, and 60 were singleton variable. The haplotype diversity of total and most sampled population was very high, however, the nucleotide diversity of every sampled population was low (Table 2). Among the total haplotypes, 82.09% are private haplotypes (Table 1).

**Table 2:** Genetic diversity of each population of *S. ningshanensis*.

Population	Haplotype diversity ±*S.D*.	Mean number of pairwise differences ±*S.D*.	Nucleotide diversity ±*S.D*.
hby	0.9591±0.0359	20.187135±9.337776	0.012309±0.006363
lfy	0.9917±0.0254	14.416667±6.820745	0.008791±0.004658
nsc	1.0000±0.0243	8.876190±4.336499	0.005412±0.002965
ljs	0.8498±0.0426	1.928854±1.137129	0.001176±0.000773
bys	0.7500±0.1071	2.091667±1.231968	0.001275±0.000841
srs	0.9333±0.0773	3.044444±1.729647	0.001856±0.001193
Total population	0.9825±0.0055	41.822511±18.302675	0.025502±0.012361

*S.D*., standard deviation.

### Phylogenetic relationships among haplotypes

**Figure 3:**
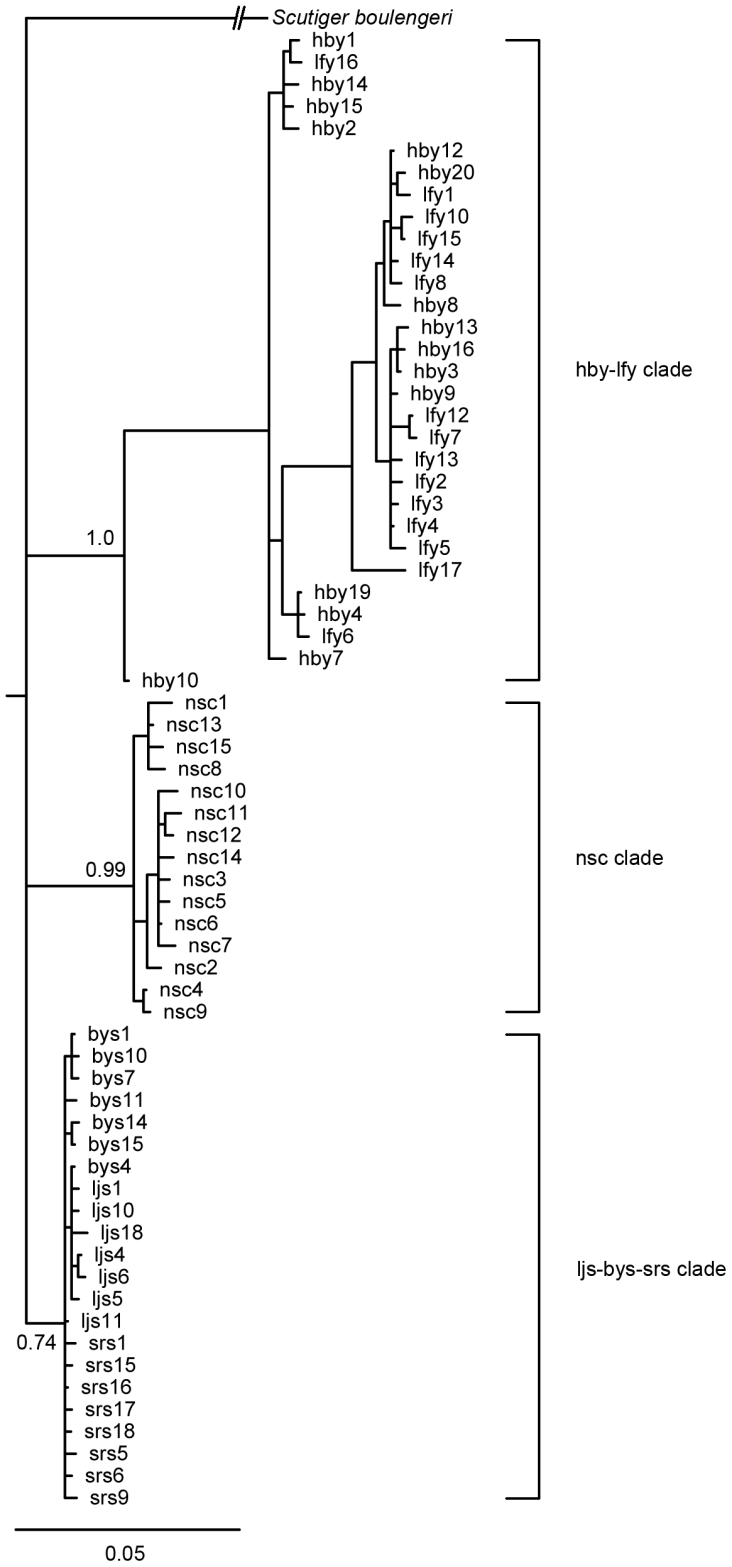
Bayesian tree for the 67 sampled haplotypes of *S*. *ningshanensis* based on the combined mtDNA cyt b and COI sequences. The Bayesian posterior probabilities from Bayesian analyses are presented above or under the main branches. The scale bar represents substitutions per site.

BI analysis revealed three distinct clades (lineages) (hby-lfy, nsc, ljs-bys-srs) in *S. ningshanensis*. Clades hby-lfy and nsc are well supported with posterior probabilities of 1 and 0.99. On the other hand clade ljs-bys-srs only has a posterior probability of 0.74 (Fig. 3). An important feature of these trees was that the components of each clade showed a strong geographical association. All haplotypes of clade hby-lfy were from western localities, all haplotypes of clade nsc were from locality nsc, all haplotypes of clade ljs-bys-srs were from the eastern localities (Fig. 1).

### Population structure and genetic differentiation

AMOVA analysis suggested that majority of the variation occurred among populations (82.15%) while differentiation within populations only contributed 17.85% to the total (Table 3). The fixation index, a measure of population differentiation due to genetic structure, was highly indicating a significant genetic differentiation among populations (*p*-value  = 0.0±0.0) (Table 3).

**Table 3:** Results of analysis of molecular variance (AMOVA) of *S. ningshanensis*.

Source of variation	*d.f.*	Sum of squares	Variance components	Percentage of variation
Among populations	5	1646.756	19.92646 Va	82.15
Within populations	93	402.547	4.32847 Vb	17.85
Total	98	2049.303	24.25492	
Fixation Index: *F*_st_ = 0.82154 (*p*-value = 0.0±0.0)				

*d.f.*, degrees of freedom.

Population comparisons showed a significant genetic differentiation (*F*_ST_) between most local populations except the population comparison between hby and lfy (Table 4).

**Table 4:** *F*_ST_ values between populations.

Population	bys	hby	lfy	ljs	nsc	srs
bys	0.0					
hby	0.82921 (*p* = 0.0±0.0)	0.0				
lfy	0.89042 (*p* = 0.0±0.0)	0.06828 (*p* = 0.06851±0.002)	0.0			
ljs	0.38150 (*p* = 0.0±0.0)	0.84979 (*p* = 0.0±0.0)	0.90412 (*p* = 0.0±0.0)	0.0		
nsc	0.86254 (*p* = 0.0±0.0)	0.77573 (*p* = 0.0±0.0)	0.83417 (*p* = 0.0±0.0)	0.87731 (*p* = 0.0±0.0)	0.0	
srs	0.31488 (*p* = 0.0±0.0)	0.79976 (*p* = 0.0±0.0)	0.86822 (*p* = 0.0±0.0)	0.33801 (*p* = 0.0±0.0)	0.83596 (*p* = 0.0±0.0)	0.0

Mantel test results showed a significant correlation between the pairwise calculated genetic distance and pairwise calculated geographical distance (Table 5) of the populations (regression coefficient  = 0.001286, correlation coefficient  = 0.77051, *p* (r_rand_≥r_obs_)  = 0.0185<0.05), indicating the existence of isolation-by-distance pattern of genetic divergence for cyt b+COI sequence, which suggests that the distribution of genetic variation is due to geographical separation rather than natural selection. The Mantel test results provided the evidence for large-scale geographical population structure in this species.

**Table 5:** Geographical distances among populations.

Population	bys	hby	lfy	ljs	nsc	srs
bys	0.0					
hby	400.14	0.0				
lfy	368.7	38.76	0.0			
ljs	20.05	381.29	350.31	0.0		
nsc	307.33	103.54	65.98	289.6	0.0	
srs	40.36	438.94	408.07	57.77	347.22	0.0

Results from SAMOVA analysis indicated that the highest *F*_CT_ equals to 0.84285 (*p* (rand.value ≥ obs.value)  = 0.01662±0.0) when *K* = 3, showing that the most likely number of populations is three.

### Historical biogeography

**Figure 4:**
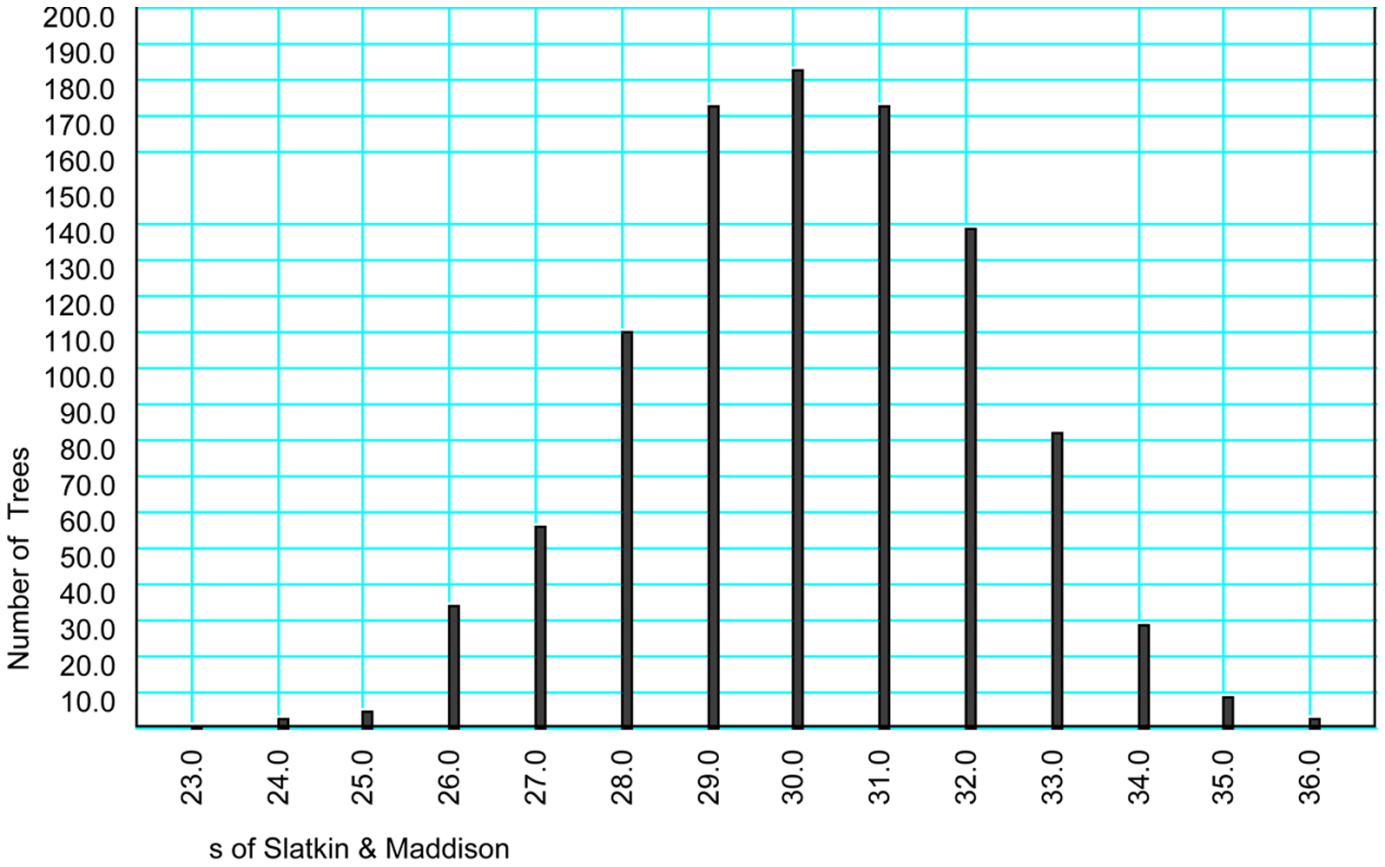
Distribution of *s*-values from simulated genealogies constrained within the models of population divergence. Single-refugium hypothesis.

The observed value of *s* is 55 which doesn't fall within the model of single-refugium indicating that the fragmentation model of population divergence was rejected (Fig. 4), that is, the current three lineages were derived from multiple refugia.

### Demographic history

**Figure 5:**
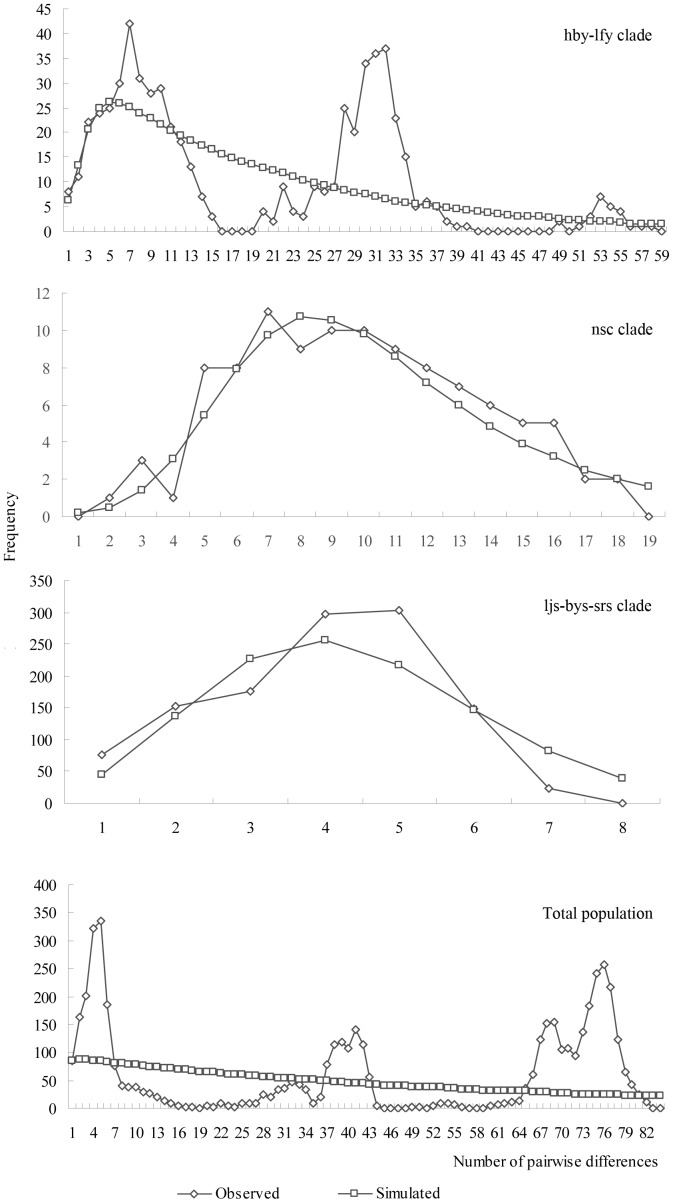
Mismatch distribution analysis for the total population and the clades.

The results of mismatch distribution showed that the *p*-values for *SSD*s and *R*s of the total population and all clades were larger than 0.05, indicating a stable population size in the past; moreover, mismatch distribution in the total population and the clade hby-lfy showed frequency distribution of pairwise difference with multiple peaks (Fig. 5), on the other hand, the *p*-values for Tajima's *D* of total population, clade hby-lfy and clade nsc were larger than 0.05 also indicating a stable population size in the past. However, the *p*-values for Fu's *F*_S_s of all clades were smaller than 0.01, and the *p*-values for Tajima's *D* of clade ljs-bys-srs was smaller than 0.05, indicating all clades experienced a sudden population expansion (Table 6); on the other hand, mismatch distribution in clades nsc and ljs-bys-srs showed frequency distribution of pairwise difference with single peak (Fig. 5), indicating an sudden population expansion in the past. The two statistics, Tajima's *D* value and the Fu's *F*_S_, are sensitive to bottleneck effects or population expansion which leads to the more negative values of Tajim's *D* and Fu's *F*_S_ [71]–[74].

**Table 6:** Mismatch distribution analyses and neutrality test of *S. ningshanensis.*

Clade	*N*	*n*	*τ* (CI = 95%)	*θ*_0_	*θ*_1_	*SSD* (*p* value)	*R* (*p* value)	Fu's *F*s (*p* value)	Tajima's *D* (*p* value)
hby-lfy	35	30	2.055 (0.0–35.352)	18.251	210.625	0.015 (0.5562)	0.005 (0.8915)	−9.08185 (0.0077)	−1.0915 (0.1265)
nsc	15	15	5.918 (2.23–16.926)	3.964	687.5	0.0024 (0.9685)	0.0082 (0.9791)	−8.12477 (0.0013)	−1.26501 (0.0931)
ljs-bys-srs	49	22	3.412 (1.396–4.701)	0.002	150.625	0.012 (0.0613)	0.044 (0.1165)	−13.343 (0.0)	−1.54633 (0.0402)
Total population	99	67	0.05859 (0.0–683.082)	53.204	99999.0	0.0219 (0.5223)	0.0044 (0.4604)	−7.80718 (0.0919)	0.64981 (0.8114)

*N*, number of sequences; *n*, number of haplotypes; *τ*, time in number of generations elapsed since the sudden expansion episode; *θ*_0_, pre-expansion, and *θ*_1_, post-expansion population size; *SSD*, sum of squared deviations; *R*, raggedness indexes.

**Figure 6:**
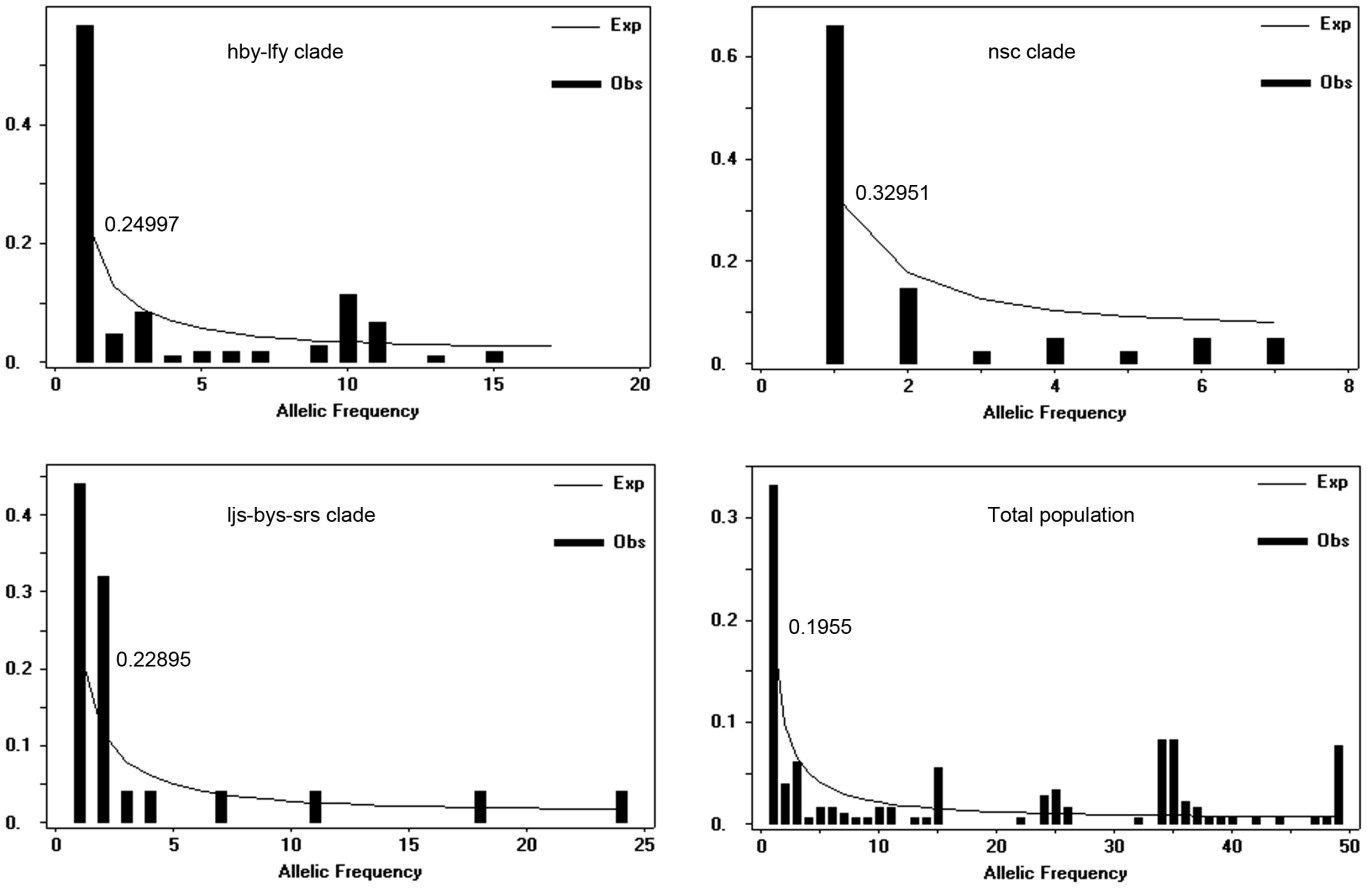
Allele frequency spectrum indicated an excess of singleton mutations in the combined mtDNA cyt b and COI sequences. Numbers above the line represent the number of sites with singleton mutations.

The allelic frequency spectrum for the entire population and all clades revealed an excess of singleton mutations and doesn't fit the neutral model (Fig. 6). The excess of singleton mutations could be caused by the expansion [58].

**Figure 7:**
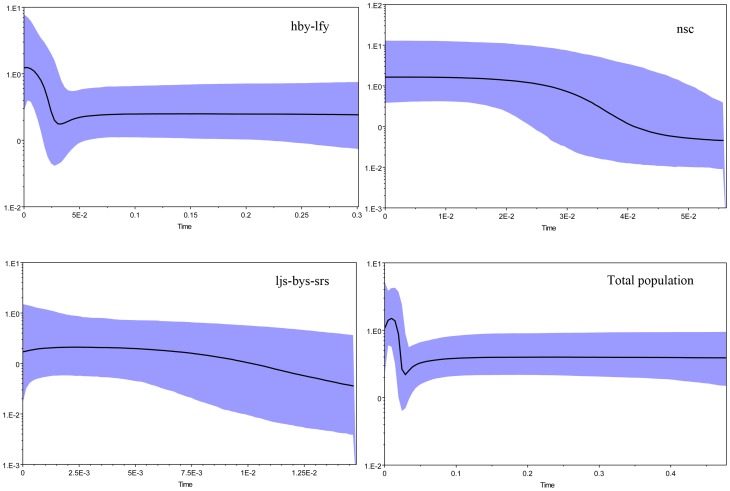
Demographic patterns of each clade and the total population as determined from the Bayesian skyline plot (BSP). The X-axis is in units of million years in the past and the Y-axis is *N*_e_*µ (effective population size × mutation rate per site per generation). The median estimates for the log10 of the population size are shown as thick solid lines, and the 95% highest posterior density (HPD) limits are shown by the shaded areas.

Bayesian skyline plot (BSP) further estimated the demographic history. The effective sample size (ESS) for all parameters of the BSP was greater than 200, showing that the 20 million generations were sufficient to determine the demographic history for each examined lineage. All lineages and the total population experienced population expansion. The hby-lfy lineage, nsc lineage and the total population experienced quick population growth after about 40,000 years ago, while the ljs-bys-srs lineage experienced a slow population growth after about 15,000 years ago. Noticeably, the hby-lfy lineage and the total population experienced a recent sharp decline in population, the ljs-bys-srs lineage showed a more recent population decline, while the nsc lineage maintained basically constant population size after the population expansion (Fig. 7).

### Genetic distances between lingeages

The lineage hby-lfy and the lineage nsc were highly divergent from each other with an uncorrected p-distances of 4.2%, the lineage hby-lfy and ljs-bys-srs were also highly divergent with an uncorrected p-distances of 4.37%. Similarly, there was an high divergence between the lineage nsc and ljs-bys-srs, with an uncorrected p-distances of 2.37%. These values were similar to those p-distances (3%) reported between two different frog species [75]. Therefore *S. ningshanensis* probably contains three cryptic species or at least three subspecies.

## Discussion

### Genetic diversity

Our results showed high levels of genetic diversity in *S. ningshanensis*. High-level genetic diversity in narrowly endemic species could be associated with the factors such as the long species history, the high-level gene flow, having experienced a recent contraction in population, multiple founder events, the maintenance of genetic diversity in refugia, the hybridization, and the ability to survive in a range of different habitats. [76]–[78]. All these factors except hybridization might contribute to the high level of genetic diversity in *S. ningshanensis*. This species has probably undergone a long history through which many mutations were accumulated since their origin. High-level gene flow is beneficial to fix the mutations in population. Multiple founder events were another alternative explanation for the high-level genetic diversity in this species. There is a possibility that multiple founder events, which accumulated more and more mutations, occurred in the history of this species. The two congeners of *S. ningshanensis* are *S*. *boulengeri* and *S. liupanensis*. The nearest localities of these two species are 167 km or 361 km respectively away from the locality hby of *S. ningshanensis*, which makes it impossible for the occurring of hybridization among these three species [79], [80]. Therefore, hybridization may not contribute to the high-level genetic diversity in *S. ningshanensis*. The distributing region of *S. ningshanensis* include a range of different habitats such as high mountains with different elevation, streams, and different vegetations. High-level diversity in habitats also contributed to the high-level genetic diversity.

### Population structure

Significant population structure occured based on the statistics pairwise differentiation. Most pairwise *F*_ST_ values are high and statistically significant. Thus, the Ningshan alpine toad appears to exhibit substantial population differentiation across the Tsinling Mountains.

Mantel test results showed a significant correlation between the genetic distance and geographical distance of the populations, indicating the presence of IBD pattern of genetic divergence for cyt b+COI sequences, suggesting that the distribution of genetic variation is due to geographical separation rather than natural selection. The Mantel test results provided the clear evidence for large-scale geographical population structure in *S. ningshanensis*. It is not possible that a significant Mantel test provided the evidence for discontinuity in the distribution of genetic variation. It rather showed a continuous distribution of the variation due to individuals mating preferentially with individuals from nearby populations [81].

AMOVA results indicated that 82.15% genetic variation occurred among populations, while differentiation within the populations only made 17.85% contributions. The high genetic variation among populations affirmed the presence of phylogeographic structure in *S. ningshanensis*. SAMOVA results and the phylogenetic relationship analysis further affirmed the existence of phylogeographic structure in this species.

Amphibians have poor dispersal capabilities and are sensitive to fine-scale landscape structure, topographic and altitudinal variation and climatic changes [82]–[88]. Many amphibians are philopatric to breeding sites [89] which restricts gene flow and leads to significant genetic differentiation among populations and lineages.

Topography and Pleistocene climate changes drive population genetic differentiation forming genetic structure pattern [90]–[92]. East Asia including China has undergone a series of cooler-drier climate cycles in the last 15 million years [93]. Dramatic climatic changes have caused the extinction of many organisms [94]. *S. ningshanensis* that distributed in the areas with low elevation disappeared during the interglacial in the Quaternary since it is an alpine species. In addition, *S. ningshanensis* retreated to a few refugia during glacial period. At the end of the Dali glaciation, *S. ningshanensis* experienced a rapid population expansion which occurred about 40,000 years before present, and it is experiencing a population contraction now probably due to anthropogenic disturbance. Topographic features, climatic fluctuation and anthropogenic activity together led to the current patchy geographic pattern for *S. ningshanensis*. This geographical distribution pattern also seen in other montane organisms [95], [96].

Finally, there is the possibility that unsampled populations between nsc and ljs may be genetically intermediate among the three groups.

### Historical biogeography

The uplifts of the Tsinling Mountains promoted genetic differentiation among lineages of *S. ningshanensis*. The Tibetan Plateau experienced three phases of rapid uplifts [97], [98]. The uplifts of the Tibetan Plateau impacted the environment of the surrounding areas including the Tsinling Mountains [97]. The Tsinling Mountains experienced similar uplift process, and three phases of uplifts occurred in Pliocene, Early Pleistocene and Holocene respectively [99]–[102]. Coalescent simulations indicated multiple refugia in *S. ningshanensis* though we could not determine the number and location of the refugia based on the statistical phylogeography analyses. Most species of the genus *Scutiger* inhabit in the eastern Tibetan Plateau [81], we guess that *S. ningshanensis* evolved when the *Scutiger* species dispersed eastward along the Tsinling Mountains. The hby-lfy lineage split apart at first, split between the lineages nsc and ljs-bys-srs occurred when this species continued dispersing from west to east on the Tsinling Mountains, while no further split occurred after the second split.

### Demographic history

Most demographic analyses revealed a sharp population expansion in all lineages of *S. ningshanensis*, and the expansion began simultaneously 40,000 years before present, which corresponds to the end of the Dali glaciation. Retreat of glacier led to population expansion in species on the Tsinling Mountains. A noteworthy phenomena is that *S. ningshanensis* is experiencing a distinct recent population contraction (though the lineage nsc is exceptional) as shown by the BSP analyses (Fig. 7), there is a possibility that anthropogenic disturbance resulted in the contraction in population size of this species. Multiple uplifts of the Tsinling Mountains and fluctuations in population size of *S. ningshanensis* associated with glacial-interglacial cycles led to increases or decreases in the levels of genetic variation and coalescence times [23], [25], [28].

### Putative cryptic species

*S. ningshanensis* probably contains at least two cryptic species or subspecies based on the p–distance analysis though there is not difference in morphology among these cryptic species or subspecies. The geographical distances between the local populations are long enough for occurrence of gene flow break in *S. ningshanensis* since it is philopatric to breeding sites. In addition, high peaks and deep valleys also contributed to the break of gene flow between populations. Subsequently, poor level of gene flow led to speciation events. However, there are limitations in taxonomic consequences based on only one taxonomic discipline. We will further confirm the cryptic speciation using data from nuclear DNA and ecological niche modeling (ENM) in the future.

### Conservation and management implications

All potential cryptic species should be considered for conservation. Different conservation strategies should be accepted for different species, therefore, it is inappropriate to protect a cryptic species complex using a single conservation strategy [103]. It is indispensable to understand and quantify biodiversity so that we can better explain and at last carry out conservation [103]. The distribution area of this species is limited to a narrow zone along the Tsinling Mountains, if this species is a cryptic species complex itself, the distribution area of each cryptic species is much smaller which increases the risk for extinction. Effective protection measures are to be provided and carried out. Conservation should be considered for every cryptic species.

Management units (MUs) are used to make conservation strategy for threatened species [104], [105]. The three lineages (hby-lfy, nsc, ljs-bys-srs) derived from our study could be used as three different MUs, each MU has its own unique genetic structure. Every MU should be considered when conservation policy is made.

## Conclusions

Three lineages were detected. The phylogenetic relationship analyses showed significant phylogeographic structure. The population expansion or contraction and genetic differentiation between populations or lineages could be explained by topography and the repetitive uplifts of the Tsinling Mountains and the climatic cycles during the late Pliocene and Pleistocene. *S. ningshanensis* experienced a rapid population expansion about 40,000 years before present. The current decline in population size was probably caused by anthropogenic disturbance. Current populations of *S. ningshanensis* are from different refugia though the location of these refugia could not be determined in our study. Topography is very important in shaping genetic connectivity. Topography, climatic changes and repetitive population expansion/contraction together led to the high level of genetic variation in *S. ningshanensis*. At least two putative cryptic species were detected. A total of three MUs were determined, which must be considered when conservation policy is made in future.
